# Lessons from zoom-university: Post-secondary student consequences and coping during the COVID-19 pandemic—A focus group study

**DOI:** 10.1371/journal.pone.0281438

**Published:** 2023-03-14

**Authors:** Anisa Morava, Anna Sui, Joshua Ahn, Wuyou Sui, Harry Prapavessis

**Affiliations:** 1 School of Kinesiology, Western University, London, ON, Canada; 2 Department of Health and Rehabilitation Sciences, Western University, London, ON, Canada; University of Johannesburg, SOUTH AFRICA

## Abstract

The COVID-19 pandemic dramatically altered the model of university education. However, the most salient challenges associated with online learning, how university students are coping with these challenges, and the impact these changes have had on students’ communities of learning remain relatively unexplored. Changes to the learning environment have also disrupted existing communities of learning for both lower and upper-year students. Hence, the purpose of our study was to explore how: (1) academic and personal/interpersonal challenges as a result of COVID-19; (2) formal and informal strategies used to cope with these academic and non-academic challenges; (3) and services or resources provided by the institution, if any, affected students’ communities of learning. Six focus groups of 5–6 students were conducted, with two focus groups specifically dedicated to upper and lower year students. Questions related to academic and interpersonal challenges, formal and informal coping strategies, and access to/use of university services/resources were posed. Common challenges included poor accommodation from professors and administrators; burnout from little separation school and personal life; lack of support for students transitioning out of university; and difficulties forming and maintaining social networks. These findings suggest the importance of fostering communities of learning informally and formally at universities beyond the pandemic context.

## Introduction

In March 2020, COVID-19 was declared a global pandemic by the World Health Organization [1]. In an attempt to mitigate the spread of the virus, numerous Canadian governing bodies imposed lockdowns and closures of non-essential services [2]. In compliance with these regulations, universities and colleges made the transition from primarily in-person instruction to hybrid or fully online learning models [3]. This educational transition, amid all the other changes to daily life as a result of the pandemic, catapulted students out of their regular routines. For example, students remaining with their families for the school year found themselves away from usual social circles while students who moved to university cities would face difficulties forming new social ties due to the social distancing mandates. The switch to online education was rife with challenges, as students, teaching assistants, and professors had to navigate online learning platforms, alter assessment strategies, and adapt delivery methods for courses that are traditionally hands-on such as labs [4].

While this transition to online learning has been largely positioned as a more accessible mode of learning, in response to and beyond the pandemic context, this has not been true for all students. Importantly, the transition to online learning disrupted previously established “communities of learning” [5]. Associated with theories such as the community of practice theory [6], which postulates engagement in a social practice is the fundamental process by which we learn—communities of learning can be defined as “a common place where people learn through group activity to define problems affecting them, to decide upon a solution, and to act to achieve the solution” [7], and have been shown to improve students’ both academic and social outcomes [8]. While research has shown that online learning can be as impactful and meaningful as in-person learning [9, 10], many other consequences of distance education have yet to be explored. Specifically, the social and interpersonal consequences to potential disruptions to communities of learning might have far-reaching effects, even as in-person learning returns.

Communities of learning have several benefits for both academic and social outcomes [11]. Leithwood and colleagues (2001) [12] highlight that the psychological sense of belonging is an important factor in student engagement and learning. Importantly, Finn & Rock (1997) [13] propose that student engagement promotes academic resilience, which can be defined as achieving favourable academic outcomes despite systemic challenges, which was crucial during the academic disruptions associated with the pandemic. Alongside the upheaval of educational routines and potential impact to communities of learning, concerns have been raised regarding the mental health of university students as a result of COVID-19, given that university students have been identified as being at a heightened risk of experiencing mental health concerns in comparison to the general public [14]. Moreover, accessing resources and services to address mental health concerns at universities remains a challenge as there is a high-volume of need and not enough service providers [15].

Although, several studies have examined the impact of COVID-19 on university students [16–19] the majority of studies have used cross-sectional or longitudinal survey designs to assess the effects of COVID-19 on several facets of student life. Elevated stress, anxiety, depression, and social isolation were commonly identified across these studies in addition to trouble concentrating and poor engagement with online learning platforms [18–20]. Research on learning disruptions during COVID-19 has primarily focused on the accessibility and acceptability of online learning [21]; however, research exploring the pandemic’s effects on communities of learning within postsecondary institutions remains sparse. Moreover, the efforts and challenges that university students have faced in order to maintain or cope with the loss of their communities of learning are not well researched.

Hence, the purpose of our study was to explore how: (1) academic and personal/interpersonal challenges as a result of COVID-19; (2) formal and informal strategies used to cope with these academic and non-academic challenges; (3) and services or resources provided by the institution, if any, affected students’ communities of learning. Our study sought to use a qualitative approach, namely focus groups, to explore the effects of COVID-19 on undergraduate students’ communities of learning at a Canadian university. Focus groups were used to gain a more nuanced understanding of the various effects of COVID-19 on several dimensions of the student experience and disruptions to student communities both in the academic and social environments, to provide the opportunity for ideas to be formed within a social context, and to center student voices across the research process [22]. Furthermore, the university where the current study took place was among a small number of Canadian universities that offered online and in-person classes (i.e., hybrid model), which may have influenced some students’ decision to remain “local” (i.e., live in the university town) or “distance” (i.e., live outside the university town [23]).

Given the contextual and experiential nature of communities of learning, our focus groups were organized specifically to explore the unique effects of the pandemic on lower-year (i.e., first- and second-year) and upper-year (i.e., third- and fourth-year). Within the context of our work, we refer to the ways in which students attempted to regain a sense of community as “coping”. We defined formal coping as the use of university-provided resources to manage the disruptions created by the COVID-19 pandemic, such as Academic Counselling, Psychological Counselling, Bursaries, Loans, and university run peer-support programs. We define informal coping as reliance on friends, family, and the strengthening of social networks for peer support and/or information.

## Methods

### Participant recruitment and eligibility

Prior to participation, each participant was provided a letter of information outlining all study procedures, as well as potential risks and benefits associated with participation, which was approved by the Western University Research Ethics Board (#118219). All study procedures were carried out in accordance with the revised version of the Helsinki Declaration (2013) [24]. Informed consent was obtained from all study participants. Recruitment posters were posted on various social media platforms (e.g., Facebook and Instagram) as well as through a mass email sent to students by the university’s MassEmailer. Interested participants were sent the letter of information and scheduled for an initial intake interview with a facilitator over Zoom. The purpose of the intake interview was to confirm eligibility, obtain verbal consent, and record demographic information along with participant availability. All intake interviews were recorded; however, confirmation of interest and verbal consent were obtained prior to any data collection.

Eligibility for this focus group included: (a) full-time enrollment in the Winter 2021 semester, (b) access to a computer, internet, and Zoom video conference software, and (c) proficiency to read, speak, and write in English.

### Focus-group facilitation guide development

The focus group facilitation guide was iteratively developed by the researchers over several sessions. Undergraduate researchers guided the development of the facilitation guide and were consulted throughout the research process about the relevance of questions, and appropriateness of questions. Following the development of the initial facilitation guide, we conducted an online pilot focus group with these undergraduates after which undergraduate researchers took part in a group debriefing where they commented and made suggestions about the facilitation guide. Through the active involvement of our undergraduate researchers, our team aimed to create a facilitation guide that was relevant and appropriate for our undergraduate focus groups.

### Procedure

Participants were assigned to groups based on their location (i.e., living in the university city or in their home city) and their year of enrolment (i.e., first- and second-year students were assigned in the lower year focus groups and third- and fourth-year students in the upper year group). All intake interviews were recorded; however, confirmation of interest and verbal consent were obtained prior to any data collection. Eligible participants were then invited to take part in one of four focus groups (5–6 participants per group) over Zoom and were emailed pre-assigned pseudonyms along with preparation instructions for the interview day (e.g., reminders that their Zoom display names would be changed to their pseudonyms, and instructions to leave their cameras off throughout the focus group). Each focus group discussion was moderated by a primary facilitator who was accompanied by a second facilitator responsible for recording the focus group and managing the time. Moderation by the primary facilitator ensured timely discussion of relevant themes and equitable division of time between participants. Moderators further probed unanticipated themes and discussion points brought up by groups members through additional questions (e.g., “I see that many of you mentioned [blank] as an effective coping mechanism and was wondering if you would be willing to expand on how [blank] works?). To begin each session, the primary facilitator reminded participants of the focus group’s purpose and procedures. To reduce the bias of socially desirable responses, facilitators requested participant confidentiality along with their honest views and opinions as there were no right or wrong answers. Participants were informed the researchers took every effort to protect their confidentiality and that they had the right to withdraw from participation at any time.

Discussion was initiated by asking participants to comment on the challenges they have faced as a result of COVID-19 (e.g., “What are some challenges that you’ve faced as an undergraduate student as a result of COVID-19?”). Discussion was continued by asking about the following topics: (a) coping mechanisms (e.g. “What are some coping mechanisms (formal or informal) you have been using this year?”), (b) awareness and means of awareness of the university’s specific resources or services (e.g. “Are you aware of any university specific resources this year?”), (c) improvements to university resources or services (e.g. “How could the university improve their current resources/services?”), and (d) recommendations for future resources or services (e.g. “Are there any resources or services you would like to see implemented in the future?”). At the end of each session, participants were given a final opportunity to add or call attention to any pertinent issues that were not addressed. Participants were then thanked for their participation with a small monetary deposit of $20 CAD (e-transferred) and emailed a debriefing letter that contained a list of resources/services available at the university. Focus groups were conducted during the months of March and April, 2021, were approximately 60 minutes in duration, audio and video recorded (via OBS Studio), and transcribed verbatim by two undergraduate researchers.

### Data analysis

Following a transcription training session, two undergraduate researchers reviewed the OBS Studio recordings and transcribed the focus groups verbatim. Three researchers subsequently reviewed the transcription to ensure the accuracy of the transcriptions and the anonymity of the participants. Proceeding initial review, the three aforementioned researchers discussed: (1) common themes, (2) the generation of initial codes (3) searching for themes and codes via NVivo (Version 11.4, 2016; QSR International Pty Ltd, Doncaster, Australia), (4) review of codes and themes, and (5) production of the final report. Specifically, two researchers conducted NVivo coding of themes independently and met with a third investigator to review, corroborate, and resolve any discrepancies in the findings. Once group consensus was achieved, the researchers worked collectively to produce the final report to ensure the accuracy and validity of the data analysis. Screen recordings were deleted after transcription as per host institution data destruction guidelines.

To achieve qualitative rigor, we relied on the criteria outlined by Guba and Lincoln (1989) [25]. First, credibility was achieved by involving students at various stages of their degrees as well as engaging in comprehensive data collection and analysis steps. To code the data, all coders read the transcripts multiple times before beginning the coding process. As well, the codes were collectively agreed on during organizational meetings prior to coding. Transferability and dependability were achieved by a third researcher who did not take part in the data collection process, but who reviewed the coded data. Confirmability was achieved by triangulation with the help of a third researcher who did not take part in the data collection process. All codes were analysed and discussed as a group following the coding process. Finally, authenticity was achieved through the presentation of verbatim quotes, even those presenting contradicting opinions. From the onset, authenticity was embedded into our research process as we deliberately sought out students at different stages of their academic careers, and developed the questionnaire with ongoing input from undergraduate students themselves.

## Results

Overall, six focus groups were conducted with a total of 32 students. Below, we present the several themes that emerged throughout our six focus group discussions (two with upper year students, two with lower; first- and second-year students, and two mixed year and location groups; see Table 1). Results are organized as “Academic Challenges” and “Personal and Interpersonal Challenges”, and as “Formal Coping” and “Informal Coping” as participants were asked to reflect on the ways in which COVID-19 has presented them with academic and personal/interpersonal challenges and how they coped with these challenges. A subsection of “Informal Coping” explores the ways in which students provided instrumental and peer support to one another through online communities and the consequences that institutional surveillance had on these spaces. Academic challenges were discussed by our participants at a much higher rate than any other concerns. Hence, we divided the academic challenges section into the following sub-headings: Institutional Failure to Accommodate Students, Shift of Learning Community Environment, Students Leaving Undergraduate Studies, Lack of Social Interaction and Connection, Formal Coping and the use of University Provided Resources, and The Use of Digital Spaces and Online Communities.

**Table 1:** Focus group demographics.

Focus Group Number	Year(s) and Location	Participants
Focus Group 1	Lower, University City	N = 6 (5 female, 1 male)
Focus Group 2	Lower, Home City	N = 5 (4 female, 1 male)
Focus Group 3	Upper, University City	N = 5 (4 female, 1 male)
Focus Group 4	Upper, Home City	N = 6 (4 female, 2 male)
Focus Group 5	Mixed, Mixed	N = 5 (4 female, 1 male)
Focus Group 6	Mixed, Mixed	N = 5 (3 female, 2 male)

### Academic challenges

#### Institutional failure to accommodate students

It is important to note that students recognized and empathized with their professors and teaching assistants, acknowledging that online teaching was strenuous, despite early efforts by universities to adequately translate existing curriculum to online and digital platforms. The students in our focus groups, both lower- and upper-year, often reflected on how difficult the learning process has been on both themselves and their teaching assistants and professors:

Kelly: “There’s kind of been this like downhill slide where the students are tired, the professors are tired like it’s hard to kind of um make a connection with your professor when you don’t get to see them.”

Interestingly, most concerns and complaints did not address the quality of the material or teaching, but rather the fractured and inadequate relationships between students and their professors and/or teaching assistants, highlighting the importance of the community aspect of learning. While lack of motivation and poor engagement were mentioned by the participants, the majority of complaints were directed at lack of or poor communication from professors or teaching assistants, failure to accommodate students, and lack of sympathy. Students drew on a fundamental disconnect between the demands of their classes and the performance expectations and expressed these difficulties in the context of social isolation:

Lily: So [the work] just felt like an extra burden on top of like a 40% midterm, and then as well as some professors have been great with being um like accommodating schedules and like extending deadlines. […] don’t realize that we have like 5 online classes and like 5 midterms that are like weighing on us, plus assignments and it can just be very overwhelming, especially when we don’t have that connection with other people.

For some of the students, lack of academic accommodation contributed to additional difficulties such as inability of the institution and professors to accommodate for students writing exams in a different time zone and an immune-compromised student who was unable to attend in-person labs, but didn’t receive accommodations through their department. Notably, this was one of the few students that identified as having a disability:

Emily: […] I do have an autoimmune condition um and I’m in a very small program and with that program it requires me to go in person. Every single day and I, you know, close contact with my professors and trying to, trying to make sure that we just keep myself like you know away from anything that may cause one stress, which is a big trigger.

The unwillingness to accommodate and scheduling conflicts often led participants to forgo volunteering obligations and club events in order to write exams as those were conducted on weekends. Familial obligations that typically took place on weekends also complicated exam and schoolwork completion. Hence, academic challenges often contributed to additional difficulties outside of an educational context by complicating other responsibilities.

#### Shift of learning community environment

Many participants admitted that they were experiencing burnout due to higher volume of work that accompanied the transition to online learning. The increased screen time as well as the increased number of assignments in lieu of exams summated to more overall screen time and more time devoted to schoolwork. For many participants, socialization could only be conducted online thereby further increasing their screen time:

Steve: I’m usually in my room in the dark doing nothing except on my computer for like, at least like 12 hours, I guess…I’m either doing work or gaming because I guess gaming is a hobby that I can do remotely. I can do it with friends. Or like friends from high school because yeah and then other then down on the computer…

Burnout was further amplified by the lack of separation between “living” space and “work” space, whereby many students occupied the same room for both work and sleep, blurring the boundary between work and leisure activities, leading to reduced motivation, boredom, and apathy:

May: Um I think one of the biggest *biggest* challenges for me is being kind of homebound even being a local student to [the institution] just I feel I’d never leave my house or where it’s like last year I left so much for class and it got me out but I just feel I’ve been so bored and unmotivated at home.

*Italics added for emphasis

Students also acknowledged that planning for daily activities became increasingly difficult as more time was spent in online learning. While asynchronous classes allowed students to watch lectures at any given time, this also made it difficult to enforce structure in daily activities:

Eve: I would say my biggest struggle so far has been having to find a way to structure my own routine because when we have in person classes you can structure your day around those and like when you’re on campus and you’re going to be back at your own place now that everything is online its harder to organize your day because there is less structure.

This lack of separation also reflected negatively on students’ sleeping patterns adding extra challenges to schoolwork. Additionally, the students in our focus groups remarked on this lack of boundary and reported more time spent on schoolwork as compared to previous years.

#### Students leaving undergraduate studies

One important theme that emerged is the disproportionate difficulty that students in “transitionary” periods unexpectedly faced as a consequence of COVID-19. These were often students who were completing their undergraduate degrees and moving on to graduate or professional school, or students who were at the threshold of important milestones within their programs. These students often felt that there was little-to-no support in their transitions and applications to graduate and professional schools. Students completing a thesis project as a degree requirement felt that they were unable to complete the projects they set out to do and instead opted out for less impactful and less exciting options in order to meet deadlines:

Kelly: […] also kind of not being able to go into the lab and not having control over the experiment or anything like that has been, um I think it just made the lab kind of run a bit slower. And for example, right now I’m about to have exam season coming up and the thesis was supposed to be done a couple of weeks ago. […] My thesis poster’s due tomorrow and I have no data and I, my thesis is due next week and I have no results, discussion, conclusion.

Some students were not able to complete a thesis project at all as a result of the COVID-19 pandemic. Students seeking out internships as part of their program requirements were unable to do so thus significantly impacting their perceived future employment prospects:

Adele: I’m in the business program here at [the institution] and recruiting is a really *really* big part. Like probably the biggest part of the program itself, and there’s like a lot of peer pressure, and the faculty also encourages us to, you know, recruit well, seek out great jobs. Uh, I would say the recruiting cycle actually starts like in the summer, so even before you enter the business program. And then it continues all the way until like, now, right? And so a lot of students are like in a constant state of stress and you know the job market is not, has not been good lately because of COVID, companies are hiring a lot less and some have been really slow to shift online and so there is a result like they buy their canceled, their programs or it’s just been really *really* difficult.

*Italics added for emphasis

Students who were graduating also found their graduation experience stressful due to poor communication from the university pertaining to graduation cut-off dates, graduation photos, and graduation requirements. Overall, students in transitionary phases felt worried they would not be able to adequately meet their future goals and were at a competitive disadvantage due to the COVID-19 pandemic.

#### Lack of social interaction and connection

Unsurprisingly, feelings of social isolation were discussed frequently in our focus groups. Given our groups were comprised of both students who resided on campus during the school year, as well as those who remained at home for the duration of their studies, we anticipated that feelings of isolation would vary depending on students’ living situations. However, even students living on campus reported feeling a loss of community. As one participant pointed out, the university had always been a student-driven community:

Adele: I honestly think that like the biggest missing piece for me was just like losing my community and I don’t really know what [the institution] could have done um because like when I think about this university, it’s always been very student driven. No matter what program you’re in, like, the students are the ones who drive like the community aspect.

Many personal and interpersonal challenges discussed in our focus groups were a direct consequence of academic challenges experienced by the students. For example, increased stress and anxiety, trouble sleeping, and a lack of activity were all a function of academic demands and changes to class format. Students also expressed concerns that they would not be able to maintain social connections they had in the past given their reliance on digital communication this year. Fostering new social connections was also viewed as difficult due to a fundamental lack of club and social activities. Students actively engaging with others on digital platforms felt that relationships started on through these platforms were more superficial or “goal-oriented” than genuine. For early (first and second) year students especially, this lack new social connections was particularly concerning, stemming from a belief that one’s first and second years were integral for forming a social network in university:

Eve: I think that one of the biggest struggles like ending second year is that people usually describe it as being one of the years where you like meet a bunch of people and where you like, get like a better sense of who like your friend group will be in undergrad. And so, I think that like a big struggle has been not being able to really meet anyone new. Like I’ve had the opportunity to like, see people from a distance, but in terms of like actually developing new friendships, it feels like I’m missing out that way.

Students also felt that, due to the long lockdown restrictions and absence of new social connection, they were experiencing a stagnation of social skills leading to increasing difficulties in forming social connections in the future:

Bee: It’s been so long since like you’ve met anyone because like by law, you’re not allowed to meet anybody right now. And so, because of that, it’s inevitable that your social skills and your desire to go and meet people and talk to people is going to decrease. And so, when you do have to do that, it’s extremely scary to do so and like for me personally…

#### Formal coping and the use of university provided resources

Seldom did the students in our focus groups rely on university provided resources in order to cope with their challenges. While such services as Academic Counselling and Psychological Counselling were mentioned, often students expressed their reservation in using these services either due to extensive waiting times or negative experiences they have had with the service previously, which would discourage future use:

Sally: I remember there was a time where I went into academic counseling and I was like crying and I was like just really upset and the whole time I just felt like they were not really like compassionate or anything and they, they were, I guess they were kind of like trying to help. So, I found that that experience has prevented me from going to them for help because I just feel like they’re not really helpful for me, and just that experience was just really negative, so I’ve kind of associated that with um with academic counseling.

While awareness of university-provided resources was not a barrier towards access for most participants, long waiting times and inadequate capacity of the resource to manage the challenge were. Several students identified specific concerns with utilizing psych services, such as feeling uncomfortable about disclosing their issue, finding the right fit with a counsellor, and struggling with whether their issue was too minor to seek formal help.

Chad: I also the mental uh, the counseling services, the counselor and the doctor I found were very quick but like within the week or maybe even a couple days, but if anyone wants to see a psychiatrist, I was told it would be at least a three month wait. Um it ended up only being six weeks, but they said on average it takes about three months.

Interestingly, the anonymity, or rather the option to not be “seen”, on virtual counselling sessions was viewed favourably by some participants expressing their concerns about privacy during in-person counselling services:

Chad: … I know when they had counseling services in person, everyone waited in this big room. So, every, like, people knew you were there but now when it’s online, you have the option to do a phone call which they consider the more secure option or zoom.”

Overall, these interactions suggest that the affective relationships and experiences formed during service use are more important than the service *itself*, and often, one negative experience can be discouraging enough that students will not utilize the resource again.

One university-provided resource that was mentioned as helpful before and during the COVID-19 pandemic were “Sophs”. Sophs (i.e., Sophomore students) occupy several roles at the university, including working as Orientation Leaders during the move-in period at the start of the academic year and as peer mentors to first-year students living on residence. Overall, Sophs integrate new students into the institute’s social and academic environments. Our participants recalled that how “good” a Soph was depended on the relationships they formed with their assigned first-year students:

Claire: […] Sophs will sort of give just general life advice and maybe like discuss about their week, um so that’s not really academic related. A lot of the time too students ask questions about some not exactly academic, but still like university related things such as housing, summer school uhm. And like how to become like connected with upper year mentors that, that type of thing.

One of the most frequently used services cited by the participants were the Self-Reported Absences (SRAs). SRAs are an institution-provided resource that are to be used by students if an absence is “short-term and unexpected” such as for medical or compassionate reasons. A justification for an absence is not required. Students are also allowed to indicate the work that they would be missing due to their absence provided the work is worth less than 30% of the final grade. During the COVID-19 pandemic, the institution increased the number of available SRAs from two to three:

Adele: […] the key thing here is that we don’t have to add a justification prior to SRA. Like say you had a family funeral, or you were suddenly really sick. Um, you had an anxiety attack. Like you would have to justify that to your professor, and that’s kind of invasive in in many ways. And also, like what happens if it’s like sudden, right? So, it’s within like the 48 hours of that exam, so SRAs are alive like within 48 hours of your assessment, and you don’t have to give a reason […] I think SRAs are really, really good, and especially during this time. […] Like I know, it’s easy to say, oh, I can’t get this done, I’m going to [use an SRA] and have it finished. Like you know two weeks later, but there’s been a lot of like feeling of being overwhelmed, and I know other people have talked about, just like the overlapping workload and people are also involved in clubs as well.

#### The use of digital spaces and online communities

For many of our participants, digital and online spaces became sources of interpersonal connection, as well as “virtual bulletin boards” through which participants learned about events and resources that were offered by the university. Several online platforms were used to facilitate this engagement such as Discord, Facebook, WhatsApp, and Reddit. Notably, the sharing of memes and negative experiences was viewed as a positive experience in of itself by our participants by reminding them that they were not alone in their isolation and struggles due to COVID-19. Large online spaces, such as a massive (nearly 30,000 members) Facebook group colloquially known as “Must Knows”, became sites for flourishing online communities, helping students share in their mutual frustration and struggle. Importantly, anonymity did not impact how much students trusted these online interactions and related to one another:

Lily: There’s a lot of online community, communities like Facebook groups that have a lot of these students just coming together and sharing like memes and finding like humor in a situation that is like very difficult for a lot of, a lot of us… And it’s nice to know that you’re not alone in how you’re feeling, and there are, um a lot of other people experiencing the same um, emotions and hardships that you are, and a lot of those communities have been helpful.

In addition, online communities facilitated social connection even through passive interaction such as studying together on Zoom:

Patricia: […] a lot of my friends aren’t the best texters so like, it’s not like they would reply all the time. But I just tried to make like an online community for myself and study with them on zoom like me, we just be on mute, but like, just at least be with each other, even though we would be doing different things.

However, online spaces were not always viewed favourably by all participants. Some of the students recalled that online spaces could be sites of academic cheating and were sometimes surveyed by professors and teaching assistants. Hence, some students expressed apprehension in using online spaces to seek out information about courses they were taking, future courses, and other resources, even when this information was not breaking academic integrity. Several participants in our groups shared stories about other students being accused of academic cheating through online spaces:

Chad: Yeah, um I was pretty lucky this year. I didn’t have anybody sending answers to things. For this year, but I know last year, when the pandemic started my friend was writing his anatomy exam and somebody had been posting the answers on a like a giant Google something and I know they got a zero and I know they specifically got a zero.

Patricia: […] like in another course, I wasn’t even in like someone in the chat, told the prof everyone who was an A, and they all got like a zero or something. So, it was hard to trust these things, but they were again, like very helpful, but I we had to be very cautious for sure.

To combat academic cheating, the institution in our study employed digital proctoring software: Proctortrack. Students in our focus groups reported several concerns rooted in university-based surveillance and personal breaches in privacy. These were often rooted in fears of being implicated in cheating by association (simply belonging to online groups where cheating took place) and often prevented students from fully utilizing online spaces. Specifically, students were concerned about their own privacy and were hesitant to use Proctortrack as the software gained access to all of their computer including sensitive and personal information (i.e., medical, bank information):

Patricia: Right so I didn’t use Proctortrack um something similar was Zoom exam, like we would be on Zoom and the prof would look but um one of my friends actually, like got her bank account money like was taken away from and it was apparently because of Proctortrack and some like data breach, which again, it’s so scary.

Additionally, the installation of the software placed additional strains on the students’ digital needs (i.e., computer) since older computers had issues running Proctortrack such as overheating. This added to the already existing exam stress:

Anonymous: Like I know for Proctortrack, especially it would sometimes make my computer overheat and then something would pop up and say your max battery is low, it’s about to go to sleep and I would just freak out. And this would be like halfway through the exam, so I wouldn’t even be done it and additionally, for like, where grades go, like there were too many written questions and I just felt like we might not have had enough time to complete them all like thoroughly.

Importantly, online platforms were often used to learn about university provided services and formal supports offered by the institution. Information about graduate school and professional school applications were also found online. Participants often expressed gratitude to the online platforms stating that wouldn’t have otherwise learned about the services provided.

Our focus group participants remarked on how certain online groups were conceived and maintained by students just like them. While the university did attempt to create online spaces for student networking, these often “died out” suggesting that the creation and maintenance of these spaces was hinged upon user creation and a sense of personal responsibility to the online space. A sense of ownership was integral to a group’s survival:

John: …But I would say that that I’ve seen attempts of either companies or I don’t know if it’s the University or companies, but them trying to make the group chat and those always died out. It…it kind of, it kind of has to be a thing with the students themselves. Take the motivation to make the group chat and they’re completely in charge of it, and those are the ones that have lasted so far, um so yeah.

## Discussion

The purpose of our study was to explore how: (1) academic and personal/interpersonal challenges as a result of COVID-19; (2) formal and informal strategies used to cope with these academic and non-academic challenges; (3) and services or resources provided by the institution, if any, affected students’ communities of learning. We were also interested in exploring whether certain groups of students (i.e., upper- vs. lower-year students) were disproportionately affected by the transitions to online and distance learning, and hence, experienced greater disruptions to their communities of learning.

### Academic challenges

The findings from our focus groups suggest that, while academic difficulties were experienced during the shift from in-person to online learning, the biggest difficulties reported by students arose from the breakdown of instructor/student relationships, and other fracturing communications. For example, students felt that instructors, while feeling stretched too thin themselves, also showed little kindness to students who were also struggling. Our focus groups revealed that relationships and “feeling heard” were more important to our participants than strict academic content or instrumental services, highlighting the importance of the community aspect of learning.

Importantly, the above findings corroborate prior work which highlighted negative impacts on academic outcomes [20, 26, 27]. In a cross-sectional mediation study by Plakhotnik and colleagues (2021) [28], it was suggested that support provided by instructors played a mediating role between the perceived impact of COVID-19 on degree completion, which was echoed in our work as students highlighted the importance of the student instructor relationship in relation to academics. Jiang (2017) [29] highlighted that a sense of community in learning can be best promoted by both student-instructor and student-student interaction rather than student-content interaction, which is an important lesson beyond the pandemic context. Thus, shifting the focus towards fostering and facilitating relationships between service providers and students may be an important step in engaging more service users, or students, in an academic context. Prior work highlighted in numerous instances that students perceived online learning negatively [26], but it was difficult to glean what specific factors contributed to the negative experience. Our work provides novel insight into factors such as issues associated with virtual proctoring during academic assessments. Another facet of academic challenges involved the provision of academic accommodation. In an investigation by Gin et al., (2021) [30] regarding accommodations for students with disabilities during COVID-19, it was identified that over half of the students were not being adequately accommodated by the university. Lack thereof or insufficient academic accommodation was an important academic challenge voiced by students in our investigation as a result of the pandemic and a concern that persists in higher education in general. Notably, we were also interested in exploring the potential role of being an upper versus lower year student, on academic and other challenges. Regarding upper versus lower year students, it became apparent that it was more so students in “transitionary” periods that faced disproportionate challenges and this is discussed in a later section.

### Personal challenges and accessing formal coping resources

Students expressed heightened academic, personal, and mental health challenges, which is in line with prior cross-sectional and longitudinal studies [16, 17]. Interestingly, Hamza and colleagues found that students without pre-existing mental health concerns were more likely to show declining mental health, which was associated with increased social isolation among these students (2021) [16]. The challenge of social isolation in conjunction with academics and mental health was discussed by many of the students in our focus groups. When students sought support for the above issues, they often felt as if they were a burden when seeking assistance through campus administrative services such as Academic Counselling, further leading to the erosion of the sense of community. An unexpected, yet novel finding was in certain instances, resources accessibility was improved during the pandemic. Specifically, services that were able to preserve the anonymity of their users such as counselling services through digital delivery, were preferred in the online medium. This is important given that mental health is a sensitive and personal topic for many students and the stigma associated with “seeing a therapist” may discourage students from seeking help. A recent narrative review highlighted that reducing barriers and fear of social stigma via online counselling and therapy can not only reach individuals who may have never sought in-person therapy, but can also serve as an “entry point” to the mental health system overall [31]. Additionally, the process of physically attending counselling sessions may be too cognitively strenuous for students requiring these services. In this sense, the shift to the digital may provide students with a more equitable access to the services they need within the post-secondary setting.

### Student-driven community building through virtual spaces

Most participants in our study found and rebuilt student communities across virtual platforms. These platforms proved integral to not only improving students’ social engagement, but also as valuable sources of coping by either offering a form of informal peer-support or by bringing attention of services offered by the university. In a study examining the use of the social media platform TikTok by gender and sexual minority youth during COVID-19, youth found a sense of belonging through this online community, gained support, and were able to share knowledge [32]. Notably, our study suggested students identified these online communities as primary sources of information even before (but mostly during) the COVID-19 pandemic. This suggests that, by engaging with already existing student-run virtual platforms and social media, institutions can reach a broader audience and better advertise services in place to serve students.

Notably, one barrier to engaging with these virtual platforms mentioned by our participants was the institution’s surveillance of groups and chat rooms. Specifically, institutional surveillance of virtual groups was noted by our participants as the main barrier to seeking assistance from peers through these platforms. While some academic cheating does take place across these virtual platforms, we argue that the benefits from, and for, student engagement far outweigh the consequences of academic cheating. Interestingly, in an investigation by Conrad and colleagues (2022) [33], the authors detail a mobile application that was integrated with a Canadian university’s central identification service that was launched during September 2020 to facilitate social interactions during the pandemic. Not surprisingly there was limited engagement with the application, 65% of respondents reported using the application “at some point” and only 26.4% indicated they used the application in the last two weeks [33]. Our findings point to the notion that these virtual groups need to be conceived and maintained *by* students, *for* students, as reported by our participants, to ensure group longevity, sustainability, and needs alignment.

Students also pointed out that “virtual proctoring” software used by the institution during exams often increased the institute’s surveillance of the students’ personal computers. Several students remarked on severe privacy breaches as a consequence of the software (one student even had money withdrawn from her bank account) and overall increased anxiety during exams. This aligns with several voiced concerns about the controversial trading practices and privacy breaches of proctoring software such as Proctorio, Examity, and Proctortrack [34].While the use of virtual proctors should resemble simply writing an exam in person, the process feels much more invasive, perhaps, due to the proctor’s access to the students’ personal computer, personal data, and their homes. Additionally, students with older computers faced significant challenges to using proctoring software as their computers were not “fast” enough and experienced information lag and being “booted out” from virtual exam rooms; these findings are echoed in previous work conducted during the pandemic [35].

As universities transition back to in-person and hybrid teaching models, we believe that online student communities will remain a large part of the student experience and students’ communities of learning. As such, institutions need to be prepared to support such communities by creating clear policies surrounding virtual proctoring and other in-class technologies that protect student privacy and accommodate students who do not have access to newer technologies. As well, institutions need to provide students with the space and the trust to create, moderate, and maintain their online communities.

### Pandemic consequences for transitioning students

Our research has demonstrated that for many students in transitionary stages of their undergraduate careers such as students who are graduating or students just entering their degrees, disruptions to communities of learning were especially consequential. These challenges are likely to persist after the pandemic is over and/or in-person course delivery resumes. For students transitioning into employment or continuing their studies in graduate or professional school, the pandemic resulted in a learning and skill acquisition gap. Many of these gaps can be attributed to the lack of mentorship and peer support during these transitionary phases.

For many, the pandemic resulted in shifting their research programmes resulting in negating the development of important practical research skills such as working with participants or working in lab environments. For students in programs that require an internship, these setbacks may impact future employment and networking opportunities. Whereas previous students could have established networking connections through their communities, students during the pandemic were mostly independently responsible for finding placements. Similar concerns were also raised by university students in the UK and Canada (at another institution) in an investigation by Appleby and colleagues’ (2022) [26]. The authors’ highlighted students were particularly concerned and disadvantaged in terms of learning experiences and future readiness due to the cancellation of practical courses, placements, and specialized facilities [26].

For students starting out in university during the pandemic the first year felt especially isolating and could result in long-lasting social consequences. We suggest that extra efforts be made in order to increase student engagement during the return to in-person learning and to foster a sense of community that many of our participants missed. Additionally, due to online learning, which was criticized for failing to facilitate relationships between students and faculty, students may require additional avenues by which to further engage with the university’s faculty.

Importantly, engaging with online universities, such as Athabasca University, would promote the transfer and sharing of knowledge of how best to promote student-faculty engagement virtually.

### Limitations

Our study is not without limitations. We acknowledge that, while focus groups provided participants with an avenue to discuss their challenges and coping strategies, our analysis did not account for participants’ diverse social identities such as race, disability, and sexuality. As such, unique challenges faced by marginalized groups could be missing. Further investigations into the challenges faced by racialized and otherwise marginalized students during the COVID-19 pandemic is needed. While participants were encouraged to self-identify as belonging to marginalized groups if they felt comfortable to do so only a few students did. This raises questions about the deliberate recruitment of diverse populations and the students’ willingness to self-identify in discussions about institutional accommodations.

Additionally, anecdotally, most of our participants learned about the study through online platforms, which may have biased the representation of these groups in terms of usage and importance within our study. Students who do not frequent social media spaces may have missed our study recruitment posters. Finally, our methods did not include peer-checking. While we recognize that this is an important step during qualitative data analysis, we decided that peer-checking would add additional strain on our participants that did not correspond with compensation we were able to provide.

## Conclusion

This study aimed to explore the ways in which post-secondary students adapted to the disruptions to their communities of learning caused by the COVID-19 pandemic. Throughout the study, we explored how students’ communities of learning were disrupted both academically and socially, and how students used both formal and informal resources to repair these communities.

Several academic challenges were identified as arising from the disruption to communities of learning, including fractured relationships with instructors and poor communication. However, we conclude that it was the interpersonal challenges in the form of missing social connections that were paramount to COVID-19 stress. Interpersonal relationships were valued more than academic content and instrumental support in both social and academic settings suggesting that universities should focus more on fostering meaningful relationships with the student body in the post-pandemic university.

While offering greater access to formal resources (e.g., counselling) should be implemented and encouraged by institutions, the formation of student-driven online communities were reported to be valuable in mitigating social isolation and served as important informal sources of academic information. Importantly, these communities need to be student-driven, with little interference from the institutions as it has been reported to impact student engagement in these environments.
